# Comparisons of neuroinflammation, microglial activation, and degeneration of the locus coeruleus-norepinephrine system in APP/PS1 and aging mice

**DOI:** 10.1186/s12974-020-02054-2

**Published:** 2021-01-06

**Authors:** Song Cao, Daniel W. Fisher, Guadalupe Rodriguez, Tian Yu, Hongxin Dong

**Affiliations:** 1 Department of Pain Medicine, Affiliated Hospital of Zunyi Medical University, 149 Dalian Street, Zunyi, 563000 Guizhou China; 2 Guizhou Key Lab of Anesthesia and Organ Protection, Affiliated Hospital of Zunyi Medical University, 6 West Xuefu Street, Zunyi, 563002 Guizhou China; 3 Department of Psychiatry and Behavioral Sciences, Northwestern University Feinberg School of Medicine, 303 East Chicago Avenue, Chicago, IL 60611 USA; 4 Department of Psychiatry and Behavioral Sciences, University of Washington Medical Center, 1959 NE Pacific St, Seattle, WA 98195 USA

**Keywords:** Alzheimer’s disease, Locus coeruleus, Norepinephrine/noradrenaline, Microglia, Norepinephrine transporter, Noradrenergic, Dopaminergic, Spinal cord

## Abstract

**Background:**

The role of microglia in Alzheimer’s disease (AD) pathogenesis is becoming increasingly important, as activation of these cell types likely contributes to both pathological and protective processes associated with all phases of the disease. During early AD pathogenesis, one of the first areas of degeneration is the locus coeruleus (LC), which provides broad innervation of the central nervous system and facilitates norepinephrine (NE) transmission. Though the LC-NE is likely to influence microglial dynamics, it is unclear how these systems change with AD compared to otherwise healthy aging.

**Methods:**

In this study, we evaluated the dynamic changes of neuroinflammation and neurodegeneration in the LC-NE system in the brain and spinal cord of APP/PS1 mice and aged WT mice using immunofluorescence and ELISA.

**Results:**

Our results demonstrated increased expression of inflammatory cytokines and microglial activation observed in the cortex, hippocampus, and spinal cord of APP/PS1 compared to WT mice. LC-NE neuron and fiber loss as well as reduced norepinephrine transporter (NET) expression was more evident in APP/PS1 mice, although NE levels were similar between 12-month-old APP/PS1 and WT mice. Notably, the degree of microglial activation, LC-NE nerve fiber loss, and NET reduction in the brain and spinal cord were more severe in 12-month-old APP/PS1 compared to 12- and 24-month-old WT mice.

**Conclusion:**

These results suggest that elevated neuroinflammation and microglial activation in the brain and spinal cord of APP/PS1 mice correlate with significant degeneration of the LC-NE system.

## Introduction

Though Alzheimer’s disease (AD) is primarily characterized by progressive memory decline, numerous other symptoms appear during the course of the disease, including neuropsychiatric symptoms [1], chronic pain [2], and seizures [3]. Chronic pain in particular also increases with aging, but AD seems to exacerbate chronic pain both quantitatively and qualitatively [4, 5]. The cellular and biochemical mechanisms that may contribute to the severity of chronic pain in AD are not well understood. One pathological change noted in the AD brain is microglial activation in the form of disease-associated microglia (DAM), leading to aberrant expression of proinflammatory cytokines in a well-characterized pattern [6–8]. Activated microglia have a nuanced role in AD and highly depend on the disease stage as well as particular activation pattern. DAM cross-seed Aβ oligomers and plaques [9, 10] enhance tau spreading and tau-driven neurodegeneration and astrogliosis [11–13] and facilitate synapse loss [14]. While these DAM are located around the neuropathological hallmarks of AD and are well correlated with cognitive decline [15, 16], it is still unclear how DAM influence other AD symptoms. Further, DAM are not only found in neural regions classically associated with AD but also in the spinal cord, where it is unclear how they may exacerbate non-cognitive AD symptoms, such as chronic pain [17].

Though neurodegeneration in cortical and hippocampal areas has been well studied in AD, the sub-cortical locus coeruleus (LC) is actually one of the first brain regions to undergo degeneration in AD pathogenesis [18, 19]. As the main source of norepinephrine (NE) in the CNS, the LC plays critical roles in a variety of brain functions, including cognition, attention, emotion, the sleep-wake cycle, and regulation of chronic pain [20–22]. While some degeneration of LC neurons also occurs in normal aging [23, 24], an estimated 50 [25] to 60% [26] loss of LC-NE neurons is observed in AD patients, far out-pacing LC loss seen with healthy aging.

Interestingly, microglia are well equipped to respond to NE signaling by expressing α_1A_, α_2A_, β_1_, and β_2_ receptors [27, 28]. Functionally, microglia are regulated by NE [23, 24, 28, 29], as NE inhibits microglial activation and reduces pro-inflammatory factors such as IL-6 and TNF-α [27]. The mechanism of these anti-inflammatory effects seem to be through activation of β_1_ and β_2_ receptors, as pharmacological agonism of these receptors in hippocampal slice cultures reduced lipopolysaccharide (LPS)-induced microglial activation and TNF-α, IL-6, and MCP-1 production [30]. Similarly, β-adrenergic agonism in microglia reduced TNF-α, IL-6, and free radical expression induced by LPS or prostaglandin E2 and protected co-cultured cortical neurons from death [31, 32]. NE suppressed Aβ-induced cytokine and chemokine production, while induced degeneration of noradrenergic neurons increased expression of inflammatory mediators in APP-transgenic mice [23].

Though LC neuron degeneration in AD may exacerbate microglial activation, it is still unclear how this influences NE levels in the brain and spinal cord, as previous studies demonstrate conflicting results in AD patients [25, 33, 34]. In addition, most of the measures used to detect changes in NE levels depend on bulk estimates from the extracellular milieu, and it is unclear if NE levels and the resultant functional impact are regulated by second messenger signaling downstream of adrenergic G protein-coupled receptors. It is possible that dysfunction in LC-NE neurotransmission leads to downstream deficits in proper adrenergic signaling that are not captured by lower-resolution estimates of total extracellular NE measurements in bulk tissue [35].

Given that the clinical data of NE levels in the AD brain are inconsistent and that there is no animal work comparing the dynamic changes in the LC-NE systems with neuroinflammation in AD mice, we investigated how microglia and the LC-NE system change during aging and AD-related neuropathogenesis in APP/PS1 mice, a commonly used animal model of AD. Importantly, we made our comparisons across important cortical, subcortical, and spinal cord regions that may influence non-cognitive symptoms that arise during dementia, notably chronic pain.

## Materials and methods

### Animals

A total of 35 APP/PS1 mice (3- and 12-month-old) and 50 WT mice (3-, 12-, and 24-month-old, sex-matched) on a C57BL6/J background were used for this study (*n* = 6/group for immunofluorescence studies, *n* = 4/group for cytokine array, *n* = 6/group for NE level studies). The breeders of APP/PS1 mice were purchased form Jackson Laboratory (Bar Harbor, Maine, USA). Animals were given food and water ad libitum and housed on a 12-h light/dark cycle. All experiments were performed under protocols approved by the Northwestern University Animal Care and Use Committee, according to the current Guide for the Care and Use of Laboratory Animals (2011, eighth edition) and NIH guidelines for the treatment of animal subjects.

### Immunofluorescence

Euthasol® (39 mg pentobarbital sodium, 5 mg phenytoin sodium, Virbac, USA) was subcutaneously injected to anesthetize mice. When mice were in deep anesthesia, the heart was exposed, and a 25G needle was inserted into the left ventricle. Mice were perfused with phosphate-buffered saline (PBS) followed by 4% paraformaldehyde. Then, the intact brain and lumbar spinal cord were collected and fixed in 4% paraformaldehyde overnight at 4 °C, followed by dehydration in 30% sucrose solution until the tissue sank to the bottom of the bottles. After fixation and dehydration, the whole brain and spinal cord were dissected and embedded in OCT compound, and 35-μm slices were cut with a cryostat (CM1850 UV, Leica, Germany). The sections containing the brain areas, i.e., the prelimbic cortex (bregma 1.94 mm) of prefrontal cortex (PFC), anterior cingulate cortex (ACC, bregma 0.74 mm), the hippocampus (bregma − 2.06 mm) encompassing the dentate gyrus (DG) and CA3, and the L4-L6 spinal cord, were collected according to the spatial coordinates of the coronal plane in the mouse brain atlas [36] and mouse spinal cord atlas [37], respectively.

For immunofluorescence staining, brain slices were (1) washed in PBS containing 0.3% Triton and blocked for 2 h at room temperature with 1% bovine serum albumin and 2% donkey or goat serum; (2) incubated overnight at 4 °C with primary antibodies: rabbit anti-Iba-1 (1:500, 019-19741, Fujifilm Wako, Japan), rabbit anti-tyrosine hydroxylase (TH, 1:500, ab112, Abcam, UK), mouse anti-norepinephrine transporter (NET, 1:500, ab211463, Abcam), and mouse anti-Aβ42 (1:500, 05-831-I, Millipore, USA); (3) washed with PBS containing 0.3% Triton and incubated for 2 h at room temperature with anti-rabbit Alexa Fluor® 488 (111-545-003, Jackson ImmunoResearch, USA; or ab150073, Abcam) or anti-mouse Cy3 (ab97035, Abcam) secondary antibodies; and (4) washed in PBS containing 0.3% Triton before being mounted with mounting medium containing Fluoroshield (ab104139, Abcam). Immunofluorescence pictures were taken with a fluorescence microscope (80i, Nikon, Japan) and CoolSNAP DYNO CCD (Photometrics, Canada).

To quantify Iba-1, TH, NET, and Aβ42 expression, the brain and spinal cord images (magnification = × 100) were outlined with the size-standardized regions of interest (ROIs) by the Image J software (v.1.52a, NIH, USA), and the percentage of area with fluorescence was quantified using this program. In particular, the threshold was set and standardized across images to maximize true protein expression signal for quantification; then, the total pixel number of target protein were recorded, and the percentage was calculated by dividing the pixel number with the total unfiltered pixel number in the ROI. TH^+^ neurons in the LC [38] (bregma − 5.4 mm [36]) were captured under the same magnification and counted manually [39]. Three sections for each brain and spinal cord region per immunostaining marker were averaged and analyzed. To evaluate non-specific staining, incubation of sections in primary or secondary antibody were conducted for each round of staining, and the resulting images confirmed that the primary and the secondary antibodies did not cause nonspecific staining.

### Cytokine array

Twelve-month-old WT and APP/PS1 mice were anesthetized and perfused with PBS; then, the intact brain and the lumbar and sacral spinal cord segments were collected and stored at − 80 °C. A mouse cytokine array (ARY006; R&D, Minneapolis, MN, USA) was used to detect cytokine expression patterns in the brain and spinal cord, and the protocol for implementation followed manufacturer’s instructions [40]. Briefly, the left half of the brain cut along the midline and the posterior border of the bilateral cerebral cortices as well as the lumbar and sacral spinal cord segments were homogenized in 0.1 M PBS with protease inhibitor cocktail (20 μL/100 mg tissue, P8340, Sigma, Israel). Total protein content was determined with BCA assay kit (23225, Thermo Fisher, USA). Membranes containing antibody arrays were incubated for 2 h at room temperature with blocking buffer (a buffered protein with preservatives). Five hundred micrograms of protein extracts was diluted in blocking buffer and incubated with the membranes overnight at 4 °C on a shaker. Then, the membranes were washed with buffered surfactant and incubated for 3 h at room temperature with the biotinylated antibody cocktail solution. After washing, the membranes were incubated with horseradish peroxidase (HRP)-conjugated streptavidin for 2 h at room temperature and developed using the detection reagent provided by the manufacturer. Images were captured with 5 min exposure time using the ChemiDoc^TM^ system (Bio-Rad, USA). Pixel densities were further analyzed using the ImageJ software and normalized to positive controls; then, mean values were calculated.

### NE evaluation with high-sensitivity ELISA

The brain and the lumbar and sacral spinal cord were prepared as described in the cytokine array experiment. NE levels were measured with a high-sensitivity enzyme-linked immunosorbent assay (ELISA) kit (NOU39-K01, Eagle Biosciences, USA). The assays apply the competitive enzyme immunoassay technique using a monoclonal antibody specific for each monoamine and a monoamine-HRP conjugate. Standards and samples were incubated together with the corresponding monoamine-HRP conjugate in pre-coated 96-well plates for 1 h. After washing, wells were incubated with a substrate for the HRP enzyme. Finally, a stop solution was added to terminate the reaction, and the intensity of the color was measured at 450 nm using a microplate reader (FLUOstar Omega, BMG Labtech, Germany). An aliquot of tissue suspended in extraction buffer was used to quantify protein concentration, which was used to normalize NE measurements. NE content was expressed as nanogram/milligram protein.

### Statistical analysis

All statistical analyses were conducted using GraphPad Prism v.7.0 (GraphPad Software, San Diego, CA, USA). Data are expressed as mean ± standard deviation. Comparisons between APP/PS1 mice and WT mice across time were estimated with two-way ANOVA, and followed by Tukey’s post hoc multiple comparison tests. A repeated-measures ANOVA was not employed as different mice were used at each time point. Cytokine expressions were compared with multiple *t* tests with false discovery rate (FDR) correction. NE levels were compared with Student’s *t* tests. *P* < 0.05 was considered as statistically significant.

## Results

### Microglial activation increased with aging in WT and APP/PS1 mice

**Fig. 1:**
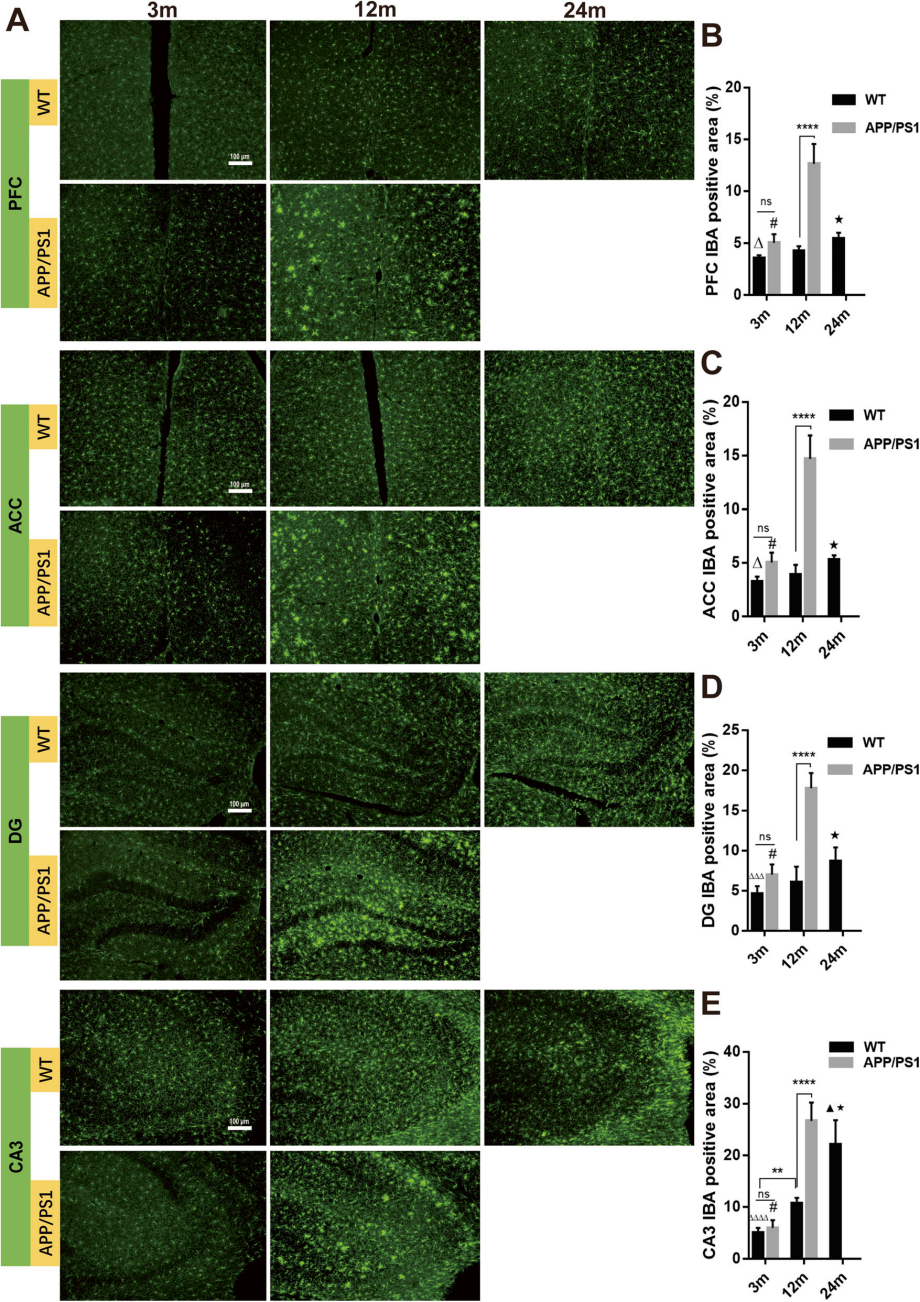
Microglia immunofluorescence of different brain areas in APP/PS1 and WT mice. **a** Immunofluorescence demonstrated that the Iba-1^+^ area in the **b** medial prefrontal cortex (PFC), **c** anterior cingulate cortex, and **d** hippocampal DG, and **e** CA3 increased significantly with aging in WT and APP/PS1 mice. In 12-month-old APP/PS1 mice, Iba-1 expression was significantly more than that of 12-month-old WT mice and 3-month-old APP/PS1 mice. In addition, in PFC, ACC, and DG, the Iba-1^+^ area in 12-month-old APP/PS1 mice was significantly more than in 24-month old WT mice. *n* = 6. ns, not significant. **P < 0.01. ****P < 0.0001. #*P* < 0.0001 compared with 12-month-old APP/PS1 mice. ▲*P* < 0.05 compared with 12-month-old WT mice. ★*P* < 0.0001 compared with 12-month-old APP/PS1 mice. Δ*P* < 0.05 compared with 24-month-old WT mice. ΔΔΔ*P* < 0.001 compared with 24-month-old WT mice. ΔΔΔΔ*P* < 0.0001 compared with 24-month-old WT mice

Activated microglia have been shown to play a critical role in AD pathogenesis, and Iba-1 expression in microglia is often used as a marker for activated microglia. To determine the dynamic changes of microglial activation across the lifespan of WT and AD mice, we compared immunofluorescent staining for Iba-1 in cortical and hippocampal regions as well as the spinal cord of WT mice at 3-, 12-, and 24-month-old WT and APP/PS1 mice at 3 and 12 months of age. Reduced survival of 24-month-old APP/PS1 mice precluded analyzing this genotype at this time point. Our data showed that in WT mice, the Iba-1^+^ area of the prelimbic prefrontal cortex (PFC), ACC, and hippocampal DG and CA3 regions increased significantly with aging (all *P* < 0.0001, Fig. 1). There were no differences in the expression of Iba-1 in all regions measured between 3-month-old APP/PS1 and WT mice, a time point before Aβ plaques were evident (all *P* > 0.05, Fig. 1a left column, Fig. 1b–e). However, there was significantly more Iba-1 expression in 12-month-old APP/PS1 in all of these regions compared to age-matched WT counterparts (all *P* < 0.0001, Fig. 1a middle column, Fig. 1b–e). Importantly, the expression of Iba-1 was more pronounced in 12-month-old APP/PS1 mice compared to 24-month-old WT mice in PFC (5.42 ± 0.23 vs 12.62 ± 0.79, *P* < 0.0001, Fig. 1b), ACC (*P* < 0.0001, Fig. 1c), DG (*P* < 0.0001, Fig. 1d), and CA3 (*P* = 0.044, Fig. 1e), suggesting AD pathogenesis has a greater effect on microglial activation than otherwise healthy aging.

**Fig. 2:**
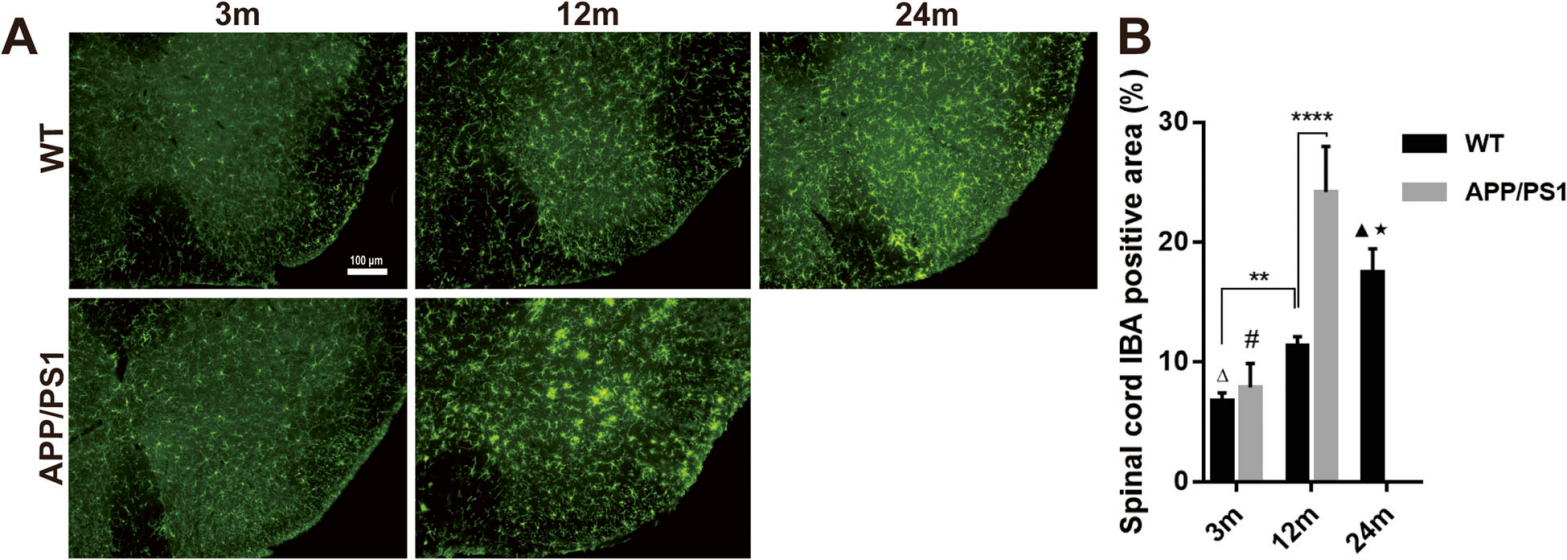
Microglia immunofluorescence in the spinal cord of APP/PS1 and WT mice. **a** Iba-1 expression in the lumbar spinal cord was detected by immunofluorescence. **b** Iba-1 expression in the two groups of mice increased significantly with aging. Iba-1^+^ area in 12-month-old APP/PS1 mice was significantly more than that of 12-month-old WT mice and 3-month-old APP/PS1 mice; it was even more than that in 24-month-old WT mice. *n* = 6. ns, not significant. **P < 0.01, ****P < 0.0001, #*P* < 0.0001 compared with 12-month-old APP/PS1 mice. Δ*P* < 0.0001 compared with 24-month-old WT mice. ▲*P* < 0.001 compared with 12 m WT mice. ★*P* < 0.0001 compared with 12 m APP/PS1 mice.

In the lumbar spinal cord, Iba-1 expression followed similar patterns to the brain, as there was increased Iba-1 expression with aging in both WT and APP/PS1 mice. Greater Iba-1 expression in AD spinal cords were detected compared to that of age-matched WT mice, and there was a greater degree of Iba-1 expression in 12-month-old APP/PS1 mice compared to 24-month-old WT mice (24.15 vs 17.47, *n* = 6, *P* < 0.0001, Fig. 2). Overall, this confirms greater microglial activation across the CNS in APP/PS1 mice.

**Fig. 3:**
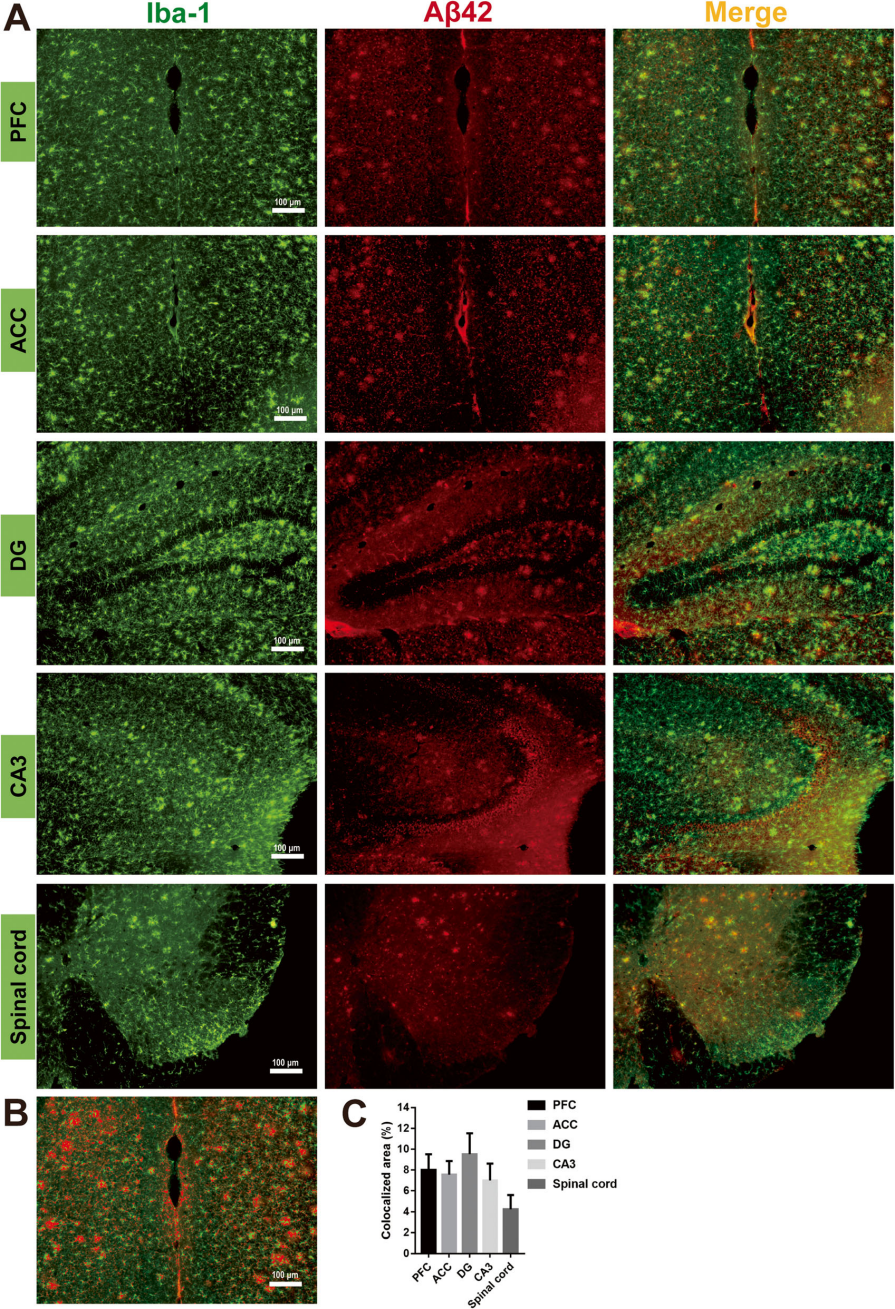
Microglia accumulating around Aβ42^+^ plaques in the brain and spinal cord. **a** Iba-1 and Aβ42 co-staining in PFC, ACC, DG, CA3, and lumbar spinal cord from 12-month-old APP/PS1 mice showed that a large number of microglia activated and accumulated around the Aβ42^+^ plaques in the brain and in the grey matter of the spinal cord. **b**, **c** Iba-1 and Aβ42 colocalized areas (yellow color in **a**) in PFC were selected and quantified with Image J

Our results suggest AD pathogenesis is a stronger driver of pathological microglial activation than aging, and so we investigated the colocalization of Aβ42 expression, a pathological hallmark of AD, and Iba-1^+^ microglia. Throughout cortical (PFC, ACC) and hippocampal regions (DG, CA3), as well as the lumbar spinal cord of 12-month-old APP/PS1 mice, increased Iba-1 and Aβ42 colocalization suggested greater recruitment of Iba-1 around Aβ42^+^ plaques (Fig. 3). Thus, we confirmed greater pathological microglial activation near Aβ42 plaques in the APP/PS1 CNS.

### Cytokine expression changed with AD status and CNS region

**Fig. 4:**
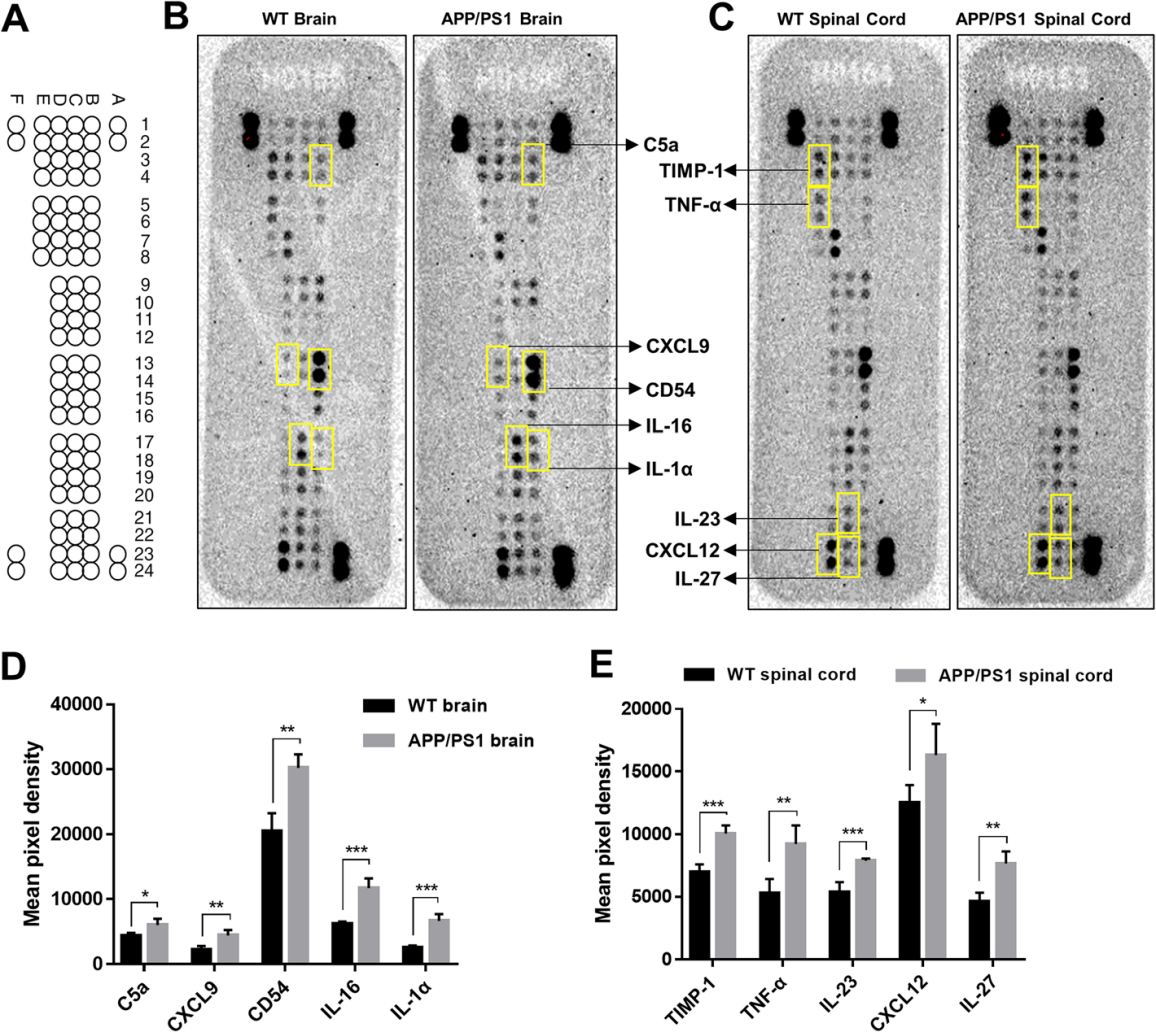
Comparisons of cytokine expression in the brain and spinal cord of WT and APP/PS1 mice. **a** Cytokine array coordinates on membranes. **b**, **c** Representative membrane images of cytokine expression in the **b** brain and **c** spinal cord. Compared with WT mice, **d** 5 cytokines were significantly upregulated in the brain of APP/PS1 mice, and **e** 5 cytokines were significantly upregulated in the spinal cord of APP/PS1 mice. *n* = 4 in each group. Statistical analyses were conducted with multiple *t* tests with false discovery rate (FDR) correction. *P < 0.05, **P < 0.01. ***P < 0.001

Activated microglia in AD could shift from protective cytokines to proinflammatory cytokines, as seen in DAM [41]. We evaluated cytokine expression in the whole brain and the lumbar and sacral spinal cord of 12-month-old WT and APP/PS1 mice using a cytokine protein array. Our results indicated that from the 40 detected cytokines, 5 cytokines (C5a, CXCL9, CD54, IL-16, and IL-1α) were upregulated (*t* = 3.1, 4.3, 5.6, 7.2, 7.3, and *P* = 0.021, 0.004, 0.001, 0.0003, 0.0003, respectively) in the brain and 5 cytokines (TIMP-1, TNF-α, IL-23, CXCL12, and IL-27) were upregulated (*t* = 6.8, 4.2, 6.0, 2.6, 5.1, and *P* = 0.0004, 0.006, 0.0009, 0.039, 0.002, respectively) in the spinal cord of APP/PS1 mice compared to age-matched WT mice (Fig. 4). These data suggest that though increased microglia lead to upregulation of particular cytokines, there may be differences in cytokine expression between the brain and spinal cord, which could lead to divergent effects on neuronal and glial homeostasis.

### LC-NE neuronal degeneration in APP/PS1 mice

**Fig. 5:**
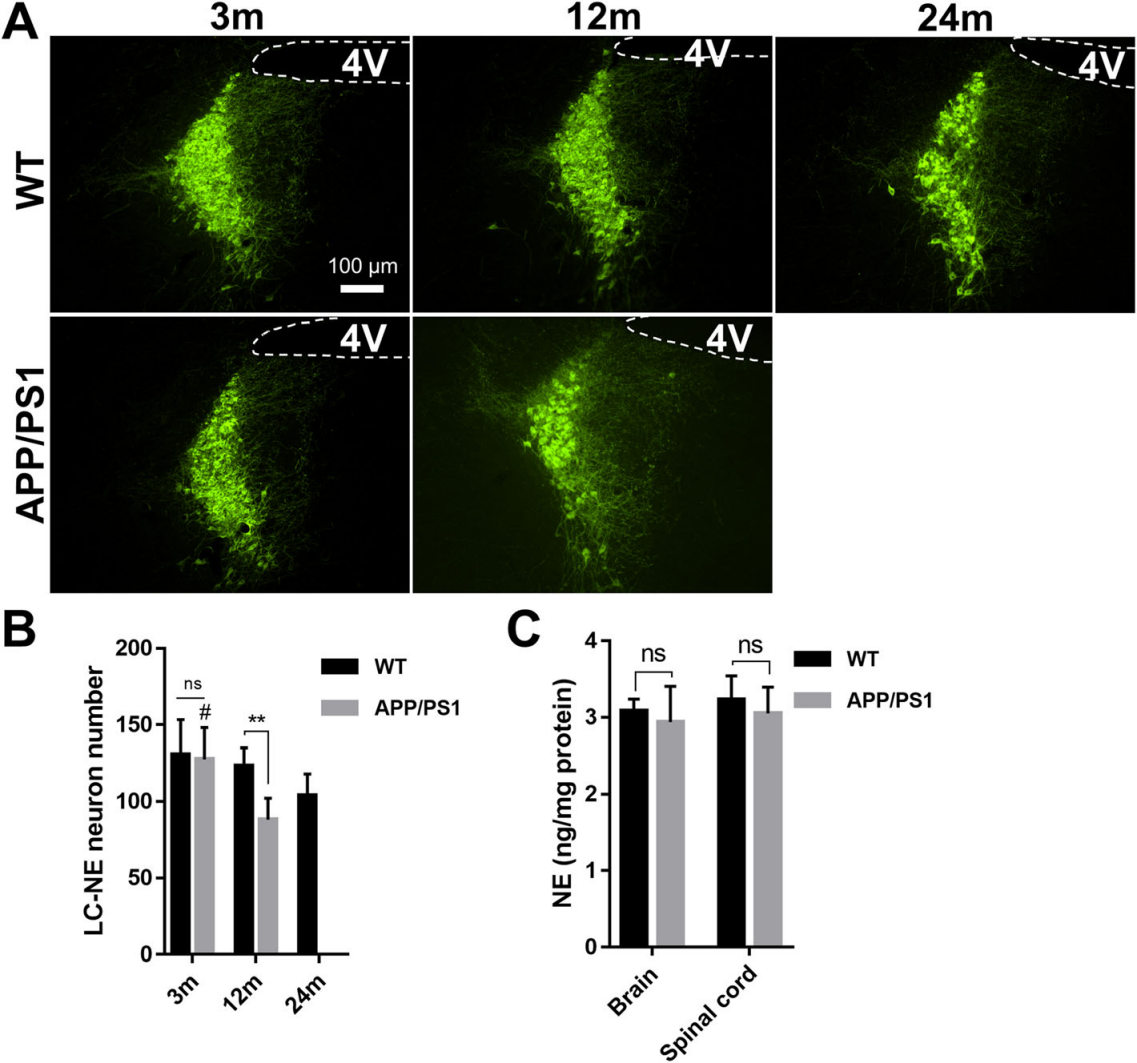
Immunofluorescence of LC-NE neuron and NE detection in the brain and spinal cord. **a** TH staining shows TH^+^ LC-NE neurons and fibers. **b** TH^+^ neurons were counted manually, and data indicated that LC-NE neurons in WT mice are gradually lost with aging, although the neuron number of 24-month-old WT mice was not significantly less than that of 3-month-old and 12-month-old WT mice. In 12-month-old APP/PS1 mice, this number decreased significantly compared to that of the 12-month-old WT mice. Three brain slices from each mouse were analyzed and averaged. *n* = 6. ns, not significant. **P < 0.01. #*P* < 0.01 compared with 12-month-old APP/PS1 mice. 4V, fourth ventricle. **c** NE level in the brain and spinal cord of APP/PS1 and WT mice was detected with ELISA. No difference was detected in brain or spinal cord between groups. *n* = 6, ns, not significant

The main source of NE in the CNS is derived from the neurons in LC, which project widely to cortical, subcortical, and spinal cord regions. As the LC is one of the first areas affected by AD, we determined the degree of LC neuron loss in WT and APP/PS1 mice at different ages using immunohistochemical analysis (Fig. 5) labeling TH. At high magnification, the TH-positive neurons are packed together, but rarely overlap. TH^+^ neuron counting indicated that LC-NE neurons in WT mice were reduced with aging (*F* = 76.36, *P* < 0.0001), although the numbers of LC-NE neurons in 24-month-old WT mice were not significantly less than that of 3-month-old (*P* = 0.06) and 12-month-old WT mice (*P* = 0.29, Fig. 5b). Additionally, the number of TH^+^ neurons in 12-month-old APP/PS1 mice was decreased compared with that of 12-month-old WT mice (123.4 ± 4.77 vs 88.18 ± 5.74, *P* = 0.0062, Fig. 5b), while LC-NE neuron numbers were similar between AD and WT mice at 3 months of age (130.8 ± 9.273 vs 127.5 ± 8.44, *P* = 0.99, Fig. 5b). Furthermore, there was more TH^+^ neuron loss in 12-month-old APP/PS1 mice as compared to 24-month-old WT mice (104 ± 5.75 vs 88.18 ± 5.74, *P* = 0.51).

### NE levels in the brain and spinal cord

As NE in the brain is mostly generated from LC neurons, and NE is likely to modulate microglial responses to neuroinflammation, we measured the NE levels in bulk tissues from the brain and spinal cord of 12-month-old AD and WT mice using ELISA. We focused on the 12-month timepoint, as LC-NE neuron number was not yet different at 3 months between the two groups. We observed no differences in NE levels in either the brain (*t* = 0.71, *P* = 0.50) or spinal cord (*t* = 0.93, *P* = 0.37) between WT and APP/PS1 mice (Fig. 5c), suggesting compensatory mechanisms are able to stabilize NE levels despite significant LC-NE neuron degeneration.

### Reduced TH^+^ nerve fibers in the brains and spinal cord of APP/PS1 mice

**Fig. 6:**
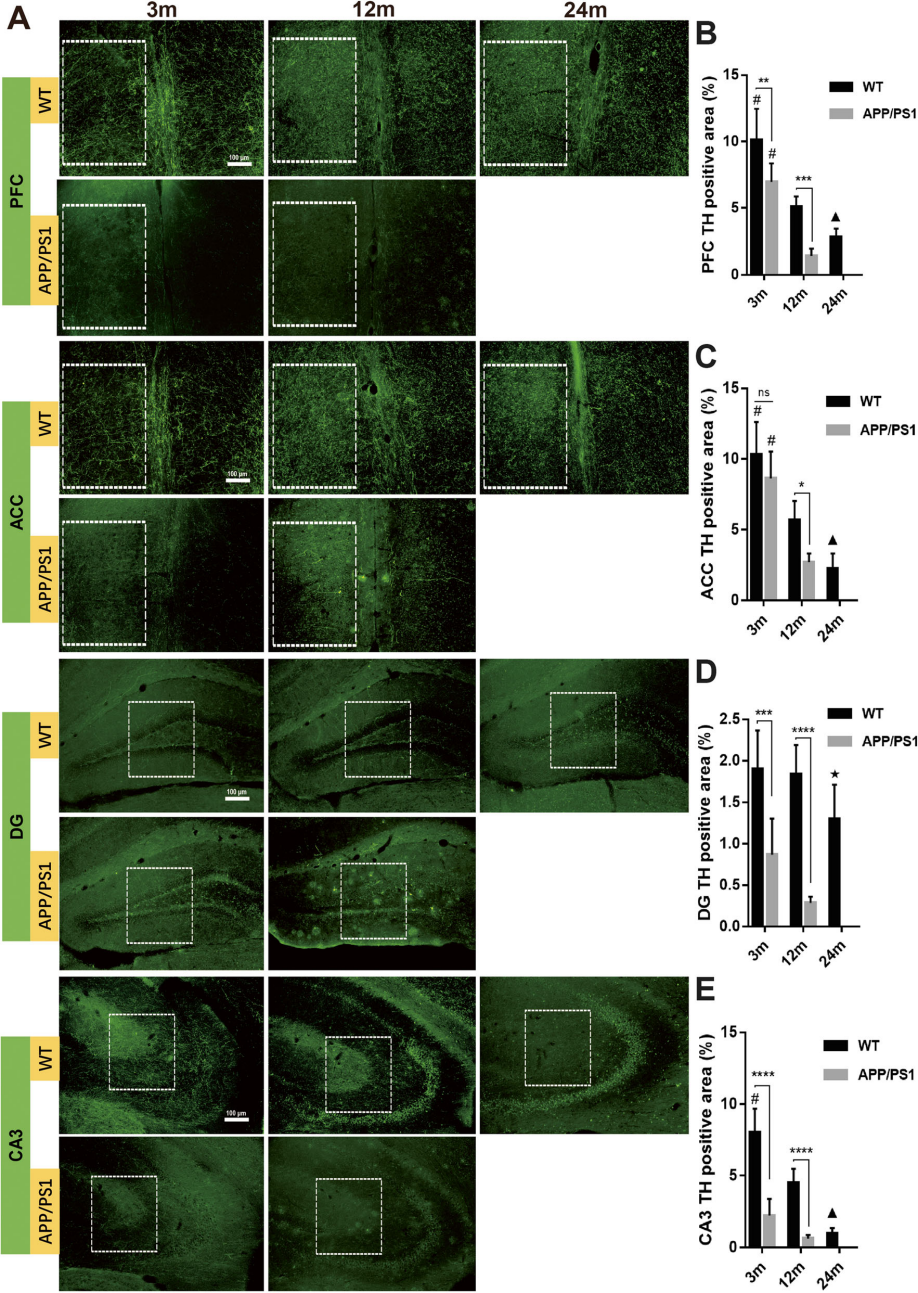
Detection of TH^+^ nerve fibers in the brains of APP/PS1 and WT mice. **a** Immunofluorescence detection of TH^+^ nerve fibers in different brain regions showed that in the **b** medial prefrontal cortex, **c** anterior cingulate, **d** DG, and **e** CA3, TH^+^ nerve fibers gradually decreased with aging. In 12-month-old APP/PS1 mice, TH^+^ area was significantly reduced compared with WT mice of the same age and 3-month-old APP/PS1 mice. TH^+^ area in the brain slices of 3-month-old and 12-month-old APP/PS1 mice was significantly lower than that of WT mice of the same age. In DG, TH^+^ area of 12-month-old APP/PS1 mice was even significantly lower than that of 24-month-old WT mice. White box indicated region of interests used for the calculation of TH^+^ area. *n* = 6. ns, not significant. *P < 0.05, **P < 0.01, ***P < 0.001, ****P < 0.0001, #*P* < 0.05 compared with other time points in the same group. ▲*P* < 0.05 compared with 12 m WT mice. ★*P* < 0.01 compared with 12 m APP/PS1 mice

It is clear that LC-NE neurons degenerated during aging and AD progression, but it has been noted that increased neurite sprouting has been detected in AD, though potentially dystrophic in nature [42]. As we detected similar levels of NE in the CNS of WT and APP/PS1 mice, we wanted to determine if increased branching of LC-NE afferents could explain these stable NE levels. TH^+^ nerve fibers reflect the axons of noradrenergic or dopaminergic neurons, and so we initially stained for these axons in cortical, hippocampal, and spinal cord regions. Immunofluorescence detection demonstrated decreased TH^+^ nerve fibers in PFC, ACC, DG, and CA3 with aging in both WT and APP/PS1 mice (Fig. 6). Similar to LC-NE degeneration, except for the ACC of 3-month-old mice (10.3% ± 2.3% vs 8.62% ± 1.9%, *P* = 0.35), 3- and 12-month-old APP/PS1 mice demonstrated reduced TH^+^ staining in the brain compared with WT mice of the same age in almost all brain regions studied (all *P* < 0.05, Fig. 6b–e). In DG (Fig. 6b), there was less TH^+^ area in 12-month-old APP/PS1 mice compared to 24-month-old WT mice (0.28% ± 0.08% vs 1.3% ± 0.41%, *P* = 0.0002, Fig. 6d), again suggesting that catecholaminergic fiber degeneration is more greatly affected by AD pathogenesis than by aging.

**Fig. 7:**
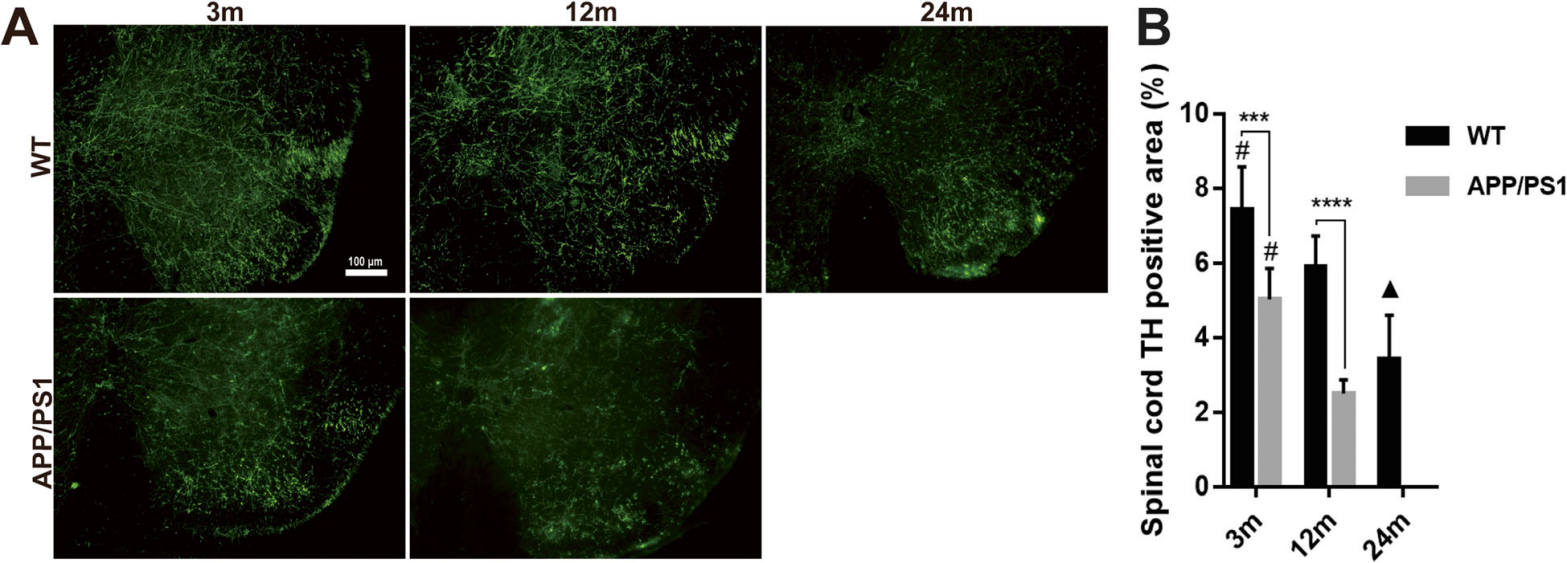
Detection of TH^+^ nerve fibers in spinal cord of APP/PS1 and WT mice. **a** TH^+^ nerve fibers in the lumbar spinal cord were detected by immunofluorescence. **b** TH^+^ fibers in the two groups of mice decreased with aging. The percentage of TH^+^ area in 3-month-old and 12-month-old APP/PS1 mice was significantly lower than that of WT mice of the same age. The TH expression in 12-month-old APP/PS1 mice was significantly less than that of 3-month-old APP/PS1 mice, and it was even less than that of 24-month-old WT mice. *n* = 6. ***P < 0.001, ****P < 0.0001, #*P* < 0.05 compared with other time points in the same group. ▲*P* < 0.05 compared with 12 m WT mice

Results in the lumbar spinal cord were similar to those in the brain: TH^+^ nerve fibers decreased with aging and AD pathogenesis (Fig. 7). Also similar to many parts of the brain, there was a greater reduction of TH^+^ nerve fibers in 3-month-old APP/PS1 mice compared to 3-month-old WT mice (7.43 ± 0.47 vs 5.02 ± 0.34, *P* = 0.0003, Fig. 7b) and in 12-month-old APP/PS1 mice compared to 12-month-old WT mice (5.9 ± 0.34 vs 2.5 ± 0.15, *P* < 0.0001, Fig. 7b), again suggesting a more deleterious effect of AD pathogenesis than aging on catecholaminergic degeneration.

### Reduced NET^+^ nerve fibers and NET expression in brains and spinal cord of APP/PS1 mice

**Fig. 8:**
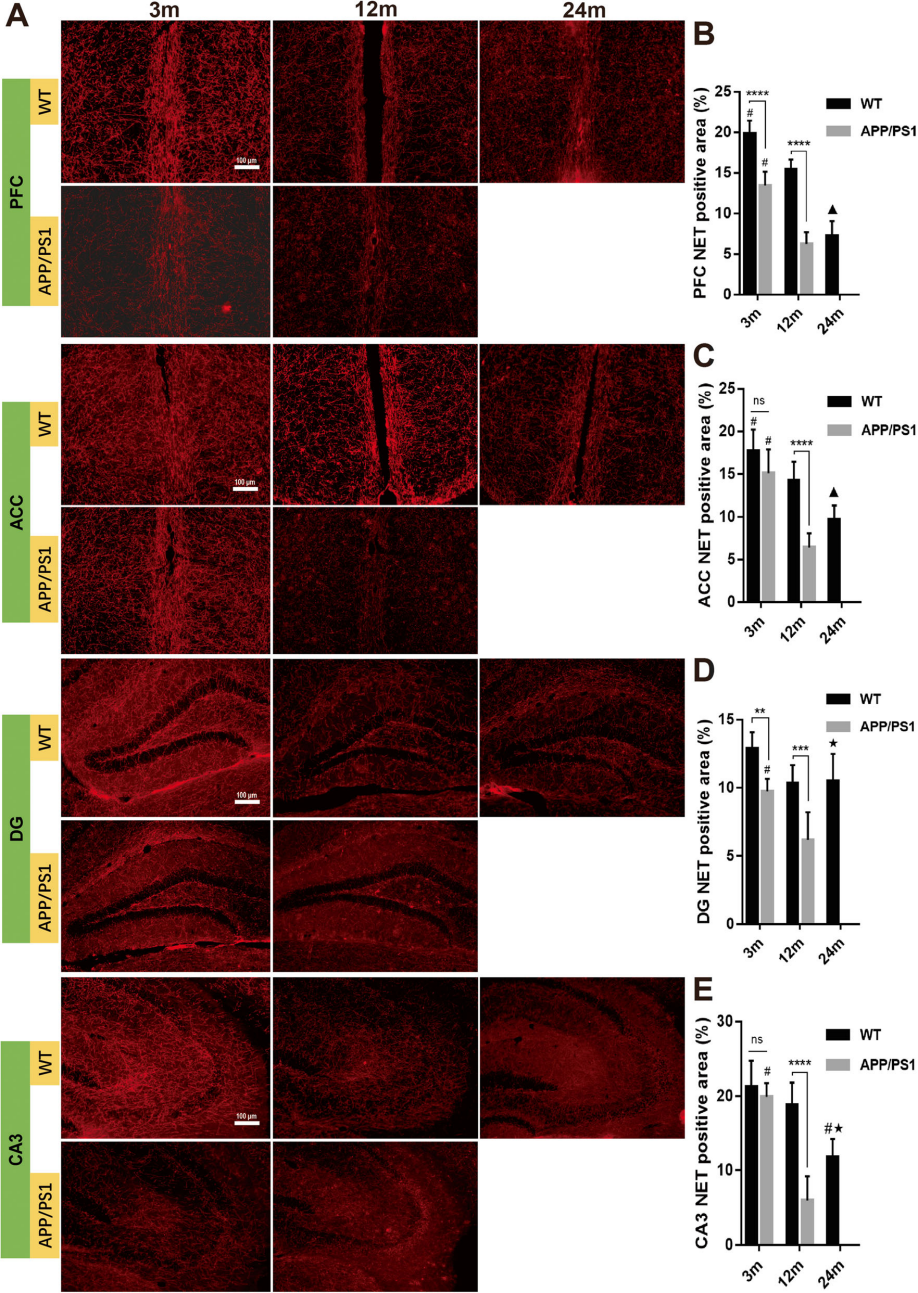
Detection of NET^+^ nerve fibers in APP/PS1 and WT mice. **a** Immunofluorescence detected NET^+^ nerve fibers in **b** medial prefrontal cortex, **c** anterior cingulate cortex, d DG, and **e** CA3. In these areas, NET^+^ nerve fibers gradually decreased with aging. NET^+^ nerve fibers in 12-month-old APP/PS1 mice were significantly less compared with WT mice of the same age and 3-month-old APP/PS1 mice. In ACC, DG, and CA3 of 12-month-old APP/PS1 mice, the NET^+^ area was less than that of 24-month-old WT mice. *n* = 6. Comparisons between APP/PS1 mice and WT mice and comparisons among 3 WT groups were estimated with two-way ANOVA and followed by Tukey’s post hoc multiple comparison tests. ns, not significant. **P < 0.01, ***P < 0.001, ****P < 0.0001, #*P* < 0.05 compared with other time points in the same group. ▲*P* < 0.05 compared with 12-month-old WT mice. ★*P* < 0.01 compared with 12-month-old APP/PS1 mice

Though TH^+^ neurons could be dopaminergic or noradrenergic, NET reflects only noradrenergic terminals. We stained for NET in APP/PS1 and WT mice to better characterize degeneration of LC-NE nerve terminals in cortical, hippocampal, and spinal cord region with aging. Our data suggest that in PFC, ACC, DG, and CA3, NET^+^ nerve fibers gradually decreased with aging (Fig. 8). NET^+^ nerve fibers in 12-month-old APP/PS1 mice were also significantly reduced compared with WT mice of the same age in PFC, ACC, DG, and CA3 (*P* < 0.0001, *P* < 0.0001, *P* < 0.001, and *P* < 0.0001, respectively, Fig. 8b–e), and a greater decrease in NET^+^ fibers was also detected in the PFC (*P* < 0.0001) and DG (*P* < 0.01) of 3-month old APP/PS1 mice. Similar to the trend of TH^+^ fibers, NET^+^ fibers were less in 12-month-old APP/PS1 mice than that in 24-month-old WT mice in DG and CA3 (*P* = 0.0002 and *P* = 0.006, respectively).

**Fig. 9:**
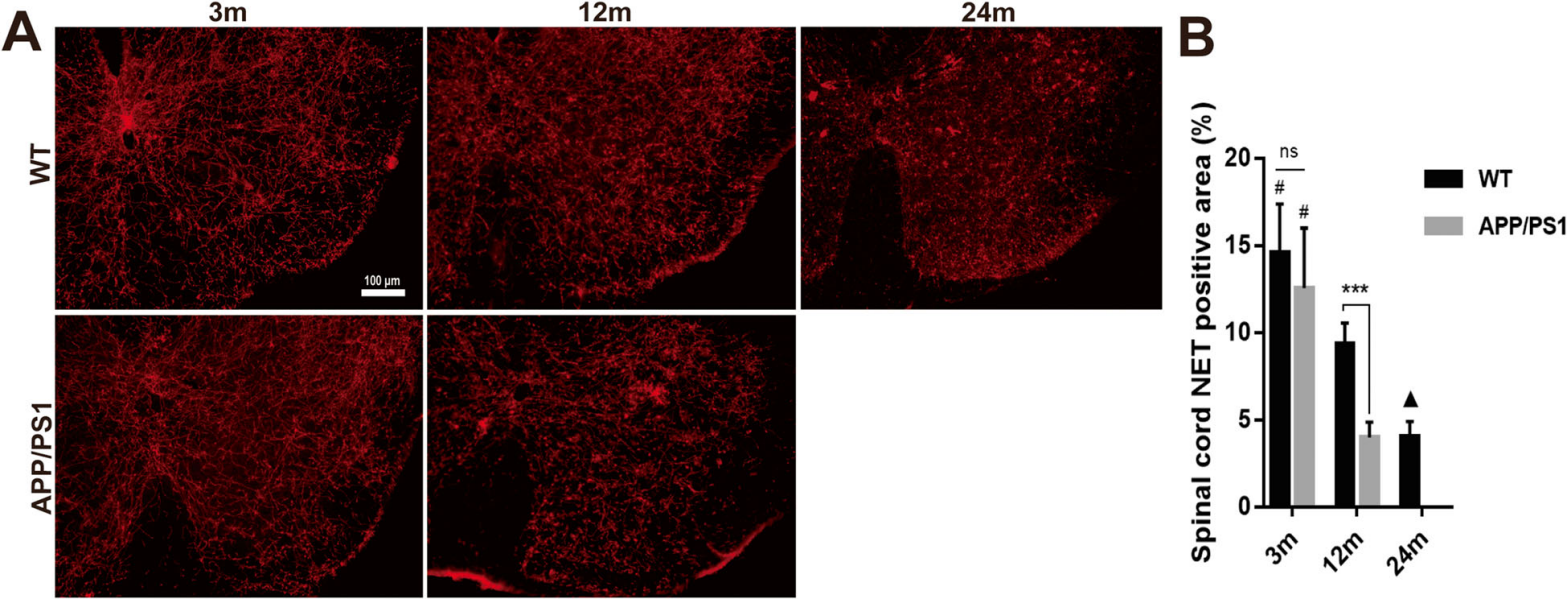
Spinal NET^+^ nerve fiber detection in APP/PS1 and WT mice. **a** The NET^+^ nerve fibers in the lumbar spinal cord were detected by immunofluorescence. **b** The NET^+^ area in the two groups of mice decreased significantly with aging. The expression of NET in 12-month-old APP/PS1 mice was significantly lower than those of WT mice of the same age and 3-month-old APP/PS1 mice. *n* = 6. ns, not significant. ***P < 0.001, #*P* < 0.05 compared with other time points in the same group. ▲*P* < 0.001 compared with 12 m WT mice

In the lumbar spinal cord of APP/PS1 and WT mice, NET^+^ fibers decreased with aging in both APP/PS1 and WT mice (*P* < 0.0001, Fig. 9). While the number of NET^+^ fibers were similar between 3-month-old WT and APP/PS1 mice (*P* = 0.46), in 12-month-old APP/PS1 mice, less NET^+^ fibers were detected compared with that of 12-month-old WT mice (9.33% ± 0.49% vs 3.98% ± 0.36%, *P* < 0.0001). However, there was no difference in NET expression levels between 12-month-old AD and 24-month-old WT mice (*P* > 0.999). Overall, this suggests that many of the reduced TH^+^ fibers were NET^+^ fibers, corroborating our observation that LC-NE degeneration is prominent in APP/PS1 mice throughout the CNS.

## Discussion

In the present study, we compared microglial activation, cytokine expression, and LC-NE neuronal degeneration in the brain and spinal cord of WT mice of 3-, 12-, and 24-months of age and APP/PS1 mice of 3- and 12-months of age, representing times before and after clear AD neuropathogenesis [43, 44]. We demonstrated that aging has a noticeable impact on both microglial activation and LC-NE degradation in WT mice. Importantly, APP/PS1 mice showed greater microglial activation and LC-NE neuronal degeneration than age-matched WT mice, which parallel with AD pathogenesis. Interestingly, microglial activation and LC-NE fiber loss in 12-month old APP/PS1 mice were even greater than in 24-month-old WT mice, suggesting that AD-like pathogenesis may have greater effects on these processes than aging. Despite these changes in LC-NE neuron number and fibers, levels of NE in brain tissue remained constant. Finally, we observed increased cytokine expression in the brain and spinal cord of APP/PS1 mice but in distinctly different patterns in these two areas, suggesting potential functional differences in the activity of microglia in these areas.

In this study, microglial activation and cytokine expression were evaluated to reflect the neuroinflammatory status of the CNS in APP/PS1 and WT mice. Microglial activation increased with aging in both APP/PS1 and WT mice; however, the severity and activation patterns were different based on genotype. Activated microglia in aged WT mice were evenly distributed in the brain and spinal cord, and number of activated microglia increased gradually with aging. However in APP/PS1 mice, activated microglia were found aggregated, particularly aggregated within and around Aβ42^+^ plaques in the brain and spinal cord at 12 months of age. Studies both in vitro [45] and in vivo [13, 46] indicate that Aβ peptides can promote microglial activation and aggregation. It has been reported that microglia are activated at an early stage of neuropathogenesis before senile plaques appear (50 days of age). Moreover, the activation of microglia remain constant in the later stages of plaque evolution (> 150 days of age) in APP/PS1 mice [47], as perhaps microglial activation is triggered by oligomeric or soluble Aβ at this early stage.

Additionally, we found that expression of several cytokines was increased in the APP/PS1 mouse brain and spinal cord. Particularly, from the 40 detected cytokines, the expression of 5 cytokines (C5a, CXCL9, CD54, IL-16, and IL-1α) in the brain and 5 cytokines (TIMP-1, TNF-α, IL-23, CXCL12, and IL-27) in the spinal cord were upregulated in APP/PS1 mice. All of these upregulated cytokines have been reported to be released by microglia, or related to microglial inflammation; for example, IL-1α and TNF-α are classically cytokine from DAM [48, 49]. Previous studies have indicated dual roles of activated microglia, one by increasing phagocytosis of Aβ, therefore decreasing Aβ plaque formation, and the other by secretion of cytokines and chemokines that can cause damage of the surrounding tissues, closer to the DAM pattern of activation seen with pathological changes [6, 50]. Our data indicated more activated microglia and upregulated cytokines in APP/PS1 mice, suggesting that more severe neuroinflammation occurred during AD pathogenesis. Heneka et al. found that NE stimulation of microglia suppressed Aβ-induced cytokine and chemokine production and increased microglial migration and phagocytosis of Aβ [23]. In APP-transgenic mice, degeneration of the LC increased expression of inflammatory mediators and resulted in elevated Aβ deposition, reduced recruitment of microglia to Aβ plaque sites, and impaired microglial Aβ phagocytosis [23]. Of note, in this assay, we only detected cytokines in bulk brain tissues and were not able to separate the cortex and hippocampus, which limited our investigation in terms of the region-specific changes. Additionally, the number of differentially expressed cytokines by the cytokine protein array was relatively small, which reflected the limitation of this screening kit. In the future, more specific analyses of brain subregions with other methods such as mass-spectroscopy are needed to compare the expression differences of inflammation-related factors in AD models.

In this study, aging in WT mice did not lead to reduction in LC-NE neuron number, even between 3-month-old and 24-month-old mice. However, we found that the loss of LC-NE neurons in APP/PS1 mice was much more evident, corroborated by Mehla et al. [51] in APP^NL-G-F/NL-G-F^ mice, a knock-in model of pathogenic APP. In 16–23-month-old female APP/PS1 mice, LC neuron number was reduced by 24% compared with non-transgenic controls [52]. Another group found that in 16–17-month-old APP/PS1 mice, LC neurons were reduced by 23%, although LC volume did not shrink [53]. In another study, LC TH^+^ neuron number was analyzed stereologically, and no difference was found between 12-month-old APP/PS1 and WT mice, although a significant decrease appeared in 18-month-old mice [54]. In another study, LC neuron volume was decreased in APP/PS1 mice at 12 months, and 18-month-old APP/PS1 mice showed decreased TH^+^ neuron volume in LC compared with 12-month-old APP/PS1 mice [54]. We detected more LC neuron loss in 12-month-old APP/PS1 mice compared with 12-month-old WT mice, and the reason for the inconsistent results among these studies may be related to the different calculation methods for the number of LC neurons and differences in experimental design.

Perhaps surprisingly, there was no significant difference in NE levels in the brain and spinal cord between 12-month-old APP/PS1 mice and WT mice despite clear reduction in LC-NE neurons with the AD-like genotype. In order to determine whether increased axonal sprouting of LC-NE neurons accounted for the stable levels of NE in the setting of profound LC-NE neuron loss, we measured the number of TH^+^ and NET^+^ nerve fibers. TH^+^ nerve fibers represent axons of noradrenergic or dopaminergic neurons, while NET is restricted to noradrenergic neurons and is not present on neurons that release dopamine [55, 56]. Our results demonstrated that compared with WT, the number of TH^+^ nerve fibers was significantly reduced in the CNS of APP/PS1 mice, including PFC, ACC, DG, CA3, and lumbar spinal cord (Figs. 6 and 7). In addition, NET^+^ nerve fibers also decreased significantly in these areas (Figs. 8 and 9). These results suggest that in APP/PS1 mice, the projection of LC-NE neurons to these brain regions is significantly decreased and NET expression is downregulated. Though it may seem contradictory that NE levels remain stable in the setting of decreased LC-NE cell bodies and nerve terminals, the reduction of NET may compensate for LC neuron degeneration. More intriguingly, this stabilization in NE levels may be part of compensatory electrophysiological changes resulting in increased excitability of LC-NE neurons, leading to greater NE release. This hyperexcitability is especially noticeable in pyramidal cells in early AD and is posited to lead to greater neurodegeneration in later AD through excitotoxic means [57]. The 12-month time point in APP/PS1 mice may represent a period where these compensatory changes are sufficient to maintain suitable NE levels while also exacerbating other pathological mechanisms that are spurring morphological degeneration. The large reduction of LC-NE nerve fibers will probably affect the amount of local NE transmitter release in its projective and distribution areas, i.e., in the synaptic and close extra-synaptic regions. As increasing evidence indicates that NE has important regulatory effects on the normal functions of microglia [24, 29], including inhibiting the activation of pro-inflammatory microglia [27, 30], it may be that these proposed more subtle changes in synaptic NE release spurs microglial activation. In support, supplying mice with the NE precursor L-threo-DOPS reduced microglial inflammation and restored microglial functions in NE-depleted mice [23]. However, this speculative decrease in synaptic NE needs to be confirmed with more sophisticated methods, though the clear reduction in LC-NE nerve terminals supports the hypothesis that regulation of microglia by LC-NE neurons is likely to be affected.

A large number of Aβ plaques and activated microglia were observed in the DG and CA3 of the hippocampus, along with concomitant reduction in TH^+^ fiber and NET^+^ in these areas. While this suggests reduced LC-NE innervation of the hippocampus, this may also indicate reduced dopamine release from catecholaminergic nerve terminals from the LC or other brain regions, as TH staining is shared by dopaminergic and noradrenergic fibers. To characterize TH^+^ fiber network in rat hippocampus, Ermine et al. used immunohistochemical and retrograde tracing techniques and found that TH and dopamine beta-hydroxylase (DβH) match well in rat brain. Especially in the rat hippocampus, all TH^+^ fibers were DβH^+^ [58]. It should be noted that although the LC has always been regarded as a noradrenergic nucleus, increasing evidence suggests that LC releases dopamine to hippocampus [58, 59] and other brain areas [60]. Dopamine also activates downstream GPCRs similar to NE and likely affects neuroinflammation and the progression of AD [61, 62]. Dopamine can also inhibit microglial activation [31, 63], similarly to NE. Therefore, the decrease in LC-NE nerve fiber projection may result in decreased dopamine release from the LC, which may further affect the activity of microglia and neuroinflammation in various brain regions innervated by the LC.

To the best of our knowledge, this is the first report of comparisons of microglial activation and noradrenergic/dopaminergic fibers in the spinal cord of APP/PS1 and WT mice. As part of the CNS, the spinal cord shows the same pathological changes as the AD brain, i.e., formation of Aβ^+^ plaques [17, 64, 65] and activated microglia around the plaques [17, 64]. In the present study, compared with WT mice, increased microglial activation, upregulated cytokine expression, and decreased noradrenergic and dopaminergic neuron projections were also evident in the spinal cords of APP/PS1 mice. These data may warrant a hypothesis whereby aggravated neuroinflammation and possible neuronal damage in the spinal cord exists in AD patients, which may change central pain processing networks in the CNS. We found that Aβ plaques in the lumbar spinal cord of 12-month-old APP/PS1 mice mainly appeared in the gray matter of the spinal cord, which is consistent with the phenomenon reported in the 5xFAD mice [64]. The spinal cord is involved in motor regulation and pain modulation, and these functions may be affected by increased spinal neuroinflammation in AD patients, as microglial activation and neuroinflammation in the spinal cord play a key role in central sensitization and maintenance of pathological pain [66].

In this study, we were unable to include APP/PS1 at 24 months of age due to the mortality issues, as it was extremely difficult to foster these mice until this age. Our hope is to continue this analysis in the future with AD mice with better survival, perhaps using the APP^NLGF^ knock-in line.

## Conclusion

Collectively, the evidence suggests that the elevated neuroinflammation in the brain and spinal cord of APP/PS1 mice is accompanied by the loss of LC-NE neuron somas and axon terminals. It is possible that this loss of LC-NE regulation further spurs pathological microglial activation, which may exacerbate central pain processing in AD patients with chronic pain.

## Data Availability

Please contact the author for data requests.

## Abbreviations

AD: Alzheimer’s disease
ACC: Anterior cingulate cortex
BPSD: Behavioral and psychological symptoms of dementia
ELISA: Enzyme-linked immunosorbent assay
DAM: Disease-associated microglia
LPS: Lipopolysaccharide
LC: Locus coeruleus
NE: Norepinephrine
NET: Norepinephrine transporter
PFC: Prefrontal cortex
TH: Tyrosine hydroxylase
WT: Wild type
